# Assessment of the general population knowledge about the emergence of Nipah virus outbreak in Bangladesh: A nationwide cross-sectional study

**DOI:** 10.1016/j.jve.2025.100585

**Published:** 2025-01-31

**Authors:** Mobin Ibne Mokbul, Shuvajit Saha, Samiha Nahar Tuli, Fatema Binte Nur, A.M. Khairul Islam, Tariful Islam, Shirsho Shreyan, Alok Bijoy Bhadra, Golam Dastageer Prince, Irfath Sharmin Eva, Mustari Nailah Tabassum, Ferdous Wahid, Md Irfan Bin Kayes, Nazim Hassan Ziad, Mohammad Delwer Hossain Hawlader

**Affiliations:** a Dhaka Medical College, Dhaka, Bangladesh; b Public Health Promotion and Development Society (PPDS), Bangladesh; c Holy Family Red Crescent Medical College, Dhaka, Bangladesh; d Icddr,b Matlab Hospital, Chandpur, Bangladesh; e Rajshahi Medical College, Rajshahi, Bangladesh; f Sheikh Hasina Medical College, Habiganj, Bangladesh; g Directorate General of Health Services, Bangladesh; h UNICEF, Bangladesh; i Chittagong Medical College, Chittagong, Bangladesh; j Dinajpur Medical College, Dinajpur, Bangladesh; k Khulna City Medical College, Khulna, Bangladesh; l Department of Public Health, School of Health and Life Sciences, North South University, Dhaka, Bangladesh; m Dhaka Medical College Research and Academic Club (DMC-RAC), Bangladesh; n Usher Institute, The University of Edinburgh, UK; o University of Rochester Medical Center, USA; p NSU Global Health Institute (NGHI), North South University, Dhaka 1229, Bangladesh

**Keywords:** Nipah virus, Bangladesh, Knowledge, Public health, Awareness, Education

## Abstract

The emergence of the Nipah virus (NiV) poses a significant global health threat, particularly in South-East Asian countries. This cross-sectional nationwide study is a pioneer in assessing knowledge levels of NiV outbreak among the general population in Bangladesh. It was conducted among the general population of Bangladesh from 15th January to 10th February 2024. A conveniently selected sample of individuals participated in the assessment of their knowledge about NiV. A semi-structured questionnaire was used as the data collection tool. After data curation, a total of 2121 responses that met the inclusion criteria were retained for analysis. Among 2121 participants, 69.38 % were aware of NiV. Overall, 62 % demonstrated good knowledge of the virus. The main sources of information were social media (29.9 %), television (25.41 %), educational institutions (18.95 %), newspapers (13.65 %), friends (6.39 %), and workplaces (5.91 %). Multivariate logistic regression analysis showed that participants aged 31–40 years had lower odds of poor knowledge (OR = 0.57, 95 % CI: 0.39–0.82, p < 0.01) compared to those aged 21–30. Females had higher odds of poor knowledge (OR = 1.38, 95 % CI: 1.05–1.81, p = 0.02) than males. Lower education levels were associated with higher odds of poor knowledge. Moreover, non-healthcare workers also had higher odds of poor knowledge compared to healthcare workers. There were regional differences, with varying odds in Rangpur (OR = 0.43, 95 % CI: 0.26–0.70, p < 0.01), Khulna (OR = 1.70, 95 % CI: 1.10–2.61, p = 0.01), and Mymensingh (OR = 2.77, 95 % CI: 1.70–4.53, p < 0.01) compared to Dhaka. The current study underscores the importance of evidence-based educational strategies, and may guide government and policymakers to design future targeted interventions to enhance public health literacy and mitigate the spread of NiV in Bangladesh as well as in its neighbouring countries.

## Graphical abstract

**Figure:**
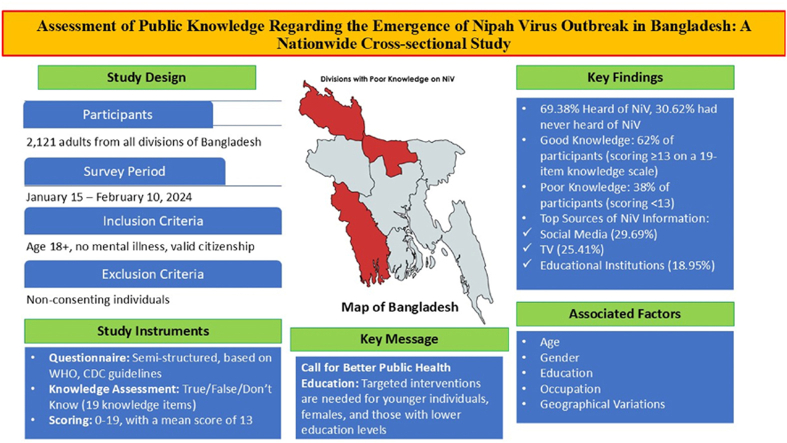

## 1 Introduction

The Nipah virus (NiV) is an emerging zoonotic paramyxovirus that poses a potential threat to global public health. It is an enveloped, non-segmented and one-stranded RNA virus of the genus Henipavirus.^1^^,^^2^ When considering the lethality and respiratory infection risks of the two strains of the virus, the NiV-B strain is more common in India and Bangladesh.^3^

The first NiV outbreak occurred in Malaysia in 1998, and since then, it has predominantly affected countries in South and Southeast Asia.^4^^,^^5^ The first Bangladeshi case was reported in the Meherpur district in May 2001, followed by Naogaon in January 2003.^6^ Since 2001, India and Bangladesh have faced almost yearly outbreaks of NiV encephalitis, underscoring the urgency of addressing this public health challenge. The majority of cases in Bangladesh were reported in the central and northwest areas. In 2024, two of the laboratory-confirmed cases died in the Dhaka division, and there have been 341 confirmed cases of NiV, including 242 deaths in Bangladesh prior to 9 February 2024.^7^ The virus had a 73 % case fatality rate until February 2023, with 8 of 11 reported cases leading to deaths, which is a stark reminder of the severity of the disease. An analysis reveals that 10 out of 11 cases had a date palm sap consumption history.^7^^,^^8^ Recently, the Institute of Epidemiology, Disease Control and Research (IEDCR) reported that NiV fatality rate had reached 100 %, with all 5 reported cases resulting in deaths.^9^

The coronavirus disease 2019 (COVID-19) pandemic has highlighted the profound global challenges posed by infectious diseases, from virus isolation to vaccine development. Similarly, the Nipah virus represents a significant public health threat, with its high mutation rate enabling it to adapt to diverse environments, infect new hosts, develop novel virulence factors, evade human immune responses, and potentially accelerate transmissibility. The adaptability and mutation rate of RNA viruses like Nipah mirror the dynamics observed during pandemics such as that of COVID-19, underscoring the urgent need for robust surveillance and proactive interventions in addressing emerging zoonotic diseases.^10^ Despite its substantial risk to human health, there remains limited understanding of NiV's genetic diversity. Pteropus bats, found across South and Southeast Asia, are known reservoirs of the virus, with serostudies detecting NiV antibodies in 3%–83 % of adult bats in the region. However, crucial aspects of NiV infection patterns within bat populations remain unclear, including the number of distinct viral lineages, their spatial distribution, and the virus ability to transmit between different Pteropus species. These gaps in knowledge underscore the urgent need for comprehensive research to inform effective public health interventions and mitigate the risk of NiV outbreaks.^11^

A survey on urban and rural health training centers in Karnataka has indicated that 36 % of rural participants considered themselves in danger of NiV infection, and compared to urban people, they were not aware of its propagation and symptoms.^12^ Research on medical interns' knowledge, attitudes, and behaviours towards NiV in Mangalore has revealed gaps in knowledge and inappropriate practices in this area^13^ There is a dearth of studies on the understanding of the general population and views on NiV infection in Bangladesh, comparable to other zoonotic diseases like COVID-19 and rabies. Research conducted in January 2021 in several villages in Bangladesh found that 58.5 % of the participants had never heard of NiV.^14^

As a result we have developed a nationwide cross-sectional study in Bangladesh to evaluate the extent of knowledge about NiV infection. This study aims to establish a baseline for subsequent interventions, to shed light on significant public health concerns, and generate new educational initiatives to equip communities to better confront NiV infection.

## 2 Methodology

### 2.1 Study design, setting, and population

This was a cross-sectional study among the general population in Bangladesh. The survey was conducted from 15th January to 10th February 2024. A conveniently selected sample of individuals was taken to measure their knowledge on NiV. Participants over 18 years of age and without mental illness were included in this study. Those without a valid document of citizenship, and not willing to participate were excluded. Before data collection, we calculated a sample size of 1991 participants considering 77.8 % prevalence generating a good level of knowledge from a previously conducted study^15^ and allocated proportionately for each division based on the census population data for 2022.^16^ A total of 2121 responses meeting our inclusion criteria were selected for analysis after data curation.

### 2.2 Study instrument

A semi-structured questionnaire was designed to be used as a tool to collect data from the participants. In order to assess the level of knowledge of NiV infection prevention and control, researchers developed the questionnaire using the Nipah virus-related guidelines published by the World Health Organization,^17^ Centers for Disease Control and Prevention,^18^ recent newspaper reports,^19^ and other previous studies.^13^^,^^15^^,^^20^, ^21^, ^22^ The questionnaire was developed in English and then translated into Bangla, face-validated, and pre-tested to determine the respondent's clarity to the survey items and for psychometric validation.

The questionnaire on NiV consisted of 25 items divided into two sections, A and B. Section A identified demographic variables and comprised 6 questions about age, gender, profession, academic qualification, and source of information about NiV. Section B included 19 statements to assess the knowledge about NiV, its mode of transmission, symptoms, prevention, and treatment.

### 2.3 Scoring

To prevent the respondents from answering correctly even if they did not really know the questionnaire was structured to answer on the knowledge scale either “true,” “false,” or “do not know.” Knowledge was assessed by giving 1 mark to a correct answer and 0 to an incorrect one or for marking ‘don't know’.

The total knowledge score ranged from a minimum of 0 (all wrong answers) to a maximum score of 19 (all correct answers) with a higher score indicating a better knowledge of NiV. In the current study, the mean knowledge score of the participants was 13. Therefore, individuals scoring ≥13 were categorised as having ‘Good Knowledge’, while those scoring <13 were categorised as having ‘Poor Knowledge’.

### 2.4 Statistical analysis

The data was analysed using R software version 4.3.3.^23^ Categorical variables were expressed as numbers and percentages, while continuous variables were presented as means and standard deviations. Univariate followed by multivariable logistic regression analyses were performed to identify the factors associated with poor knowledge. All values of p were considered statistically significant if < 0.05.

### 2.5 Ethical consideration

Ethical approval was obtained from the Institutional Review Board (IRB)/Ethical Review Committee (ERC) of the North South University, Bangladesh (IRB approval #2022IOR-NSU/lRB/1204). The questionnaire form contained a detailed explanation of the study's purpose as well as a consent form. Participation in this study was entirely voluntary. Participant privacy and data safety were strictly protected according to the IRB instructions and ethical standards of the 1964 Declaration of Helsinki and its later amendments or comparable ethical standards were followed wherever applicable.

## 3 Results

Table 1 shows the socio-demographic characteristics of the participants. The majority were in the age group of 21–30 years (41.35 %), followed by 31–40 years (24.94 %). In terms of gender distribution, with a near-equal representation, including 51.63 % male and 48.37 % female participants. Notably, the largest proportion (29.37 %) comprised graduate individuals, followed by those with higher secondary education (25.79 %). Occupationally, the majority of participants were students (28.29 %), followed by housewives (21.03 %) and service and job holders (17.92 %). The data showed regional diversity, with participants spread across various divisions of Bangladesh. Dhaka emerged as the division with the highest representation (26.97 %), followed by Chittagong (17.73 %), Rangpur (12.87 %), and Rajshahi (11.93 %).

**Table 1:** Socio-demographic characteristics of participants.

Socio-demographic characteristics	Frequency (n = 2121)	Percentage (%)
Age (Years)		
≤20	159	7.50
21–30	877	41.35
31–40	529	24.94
41–50	331	15.61
>50	225	10.61
**Gender**		
Male	1095	51.63
Female	1026	48.37
**Education**		
No formal education	149	7.02
Primary	187	8.82
Secondary	337	15.89
Higher secondary	547	25.79
Graduation	623	29.37
Postgraduate	278	13.11
**Occupation**		
Student	600	28.29
Housewife	446	21.03
Service and job holders	380	17.92
Business	332	15.65
Healthcare workers	148	6.98
Others	215	10.14
**Divisions**		
Dhaka	572	26.97
Chittagong	376	17.73
Rangpur	273	12.87
Rajshahi	253	11.93
Khulna	222	10.47
Mymensingh	190	8.96
Sylhet	123	5.80
Barishal	112	5.28

**Fig. 1:**
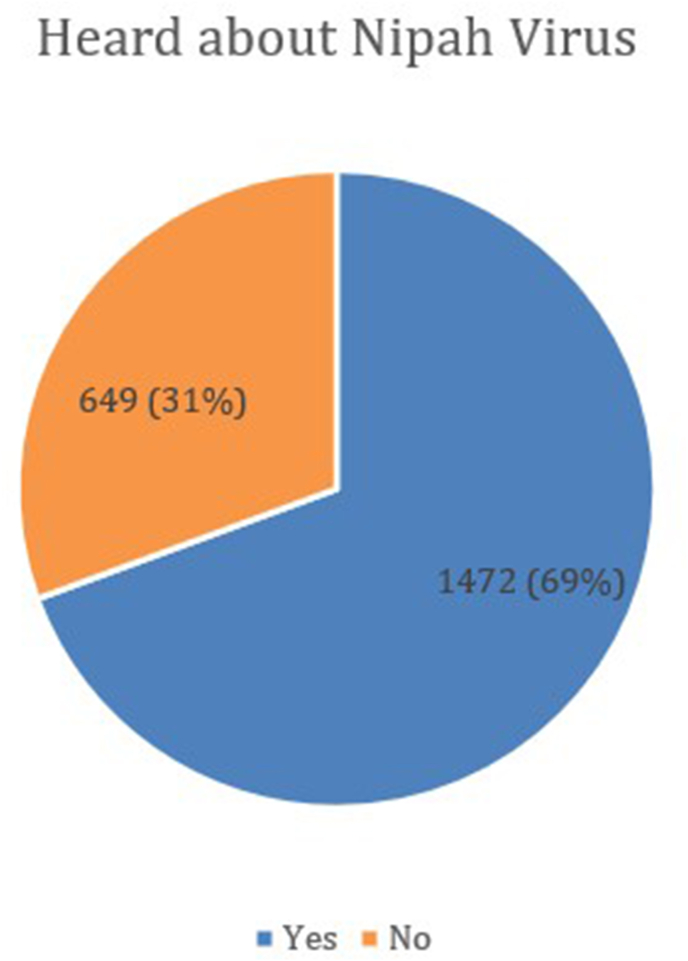
Response from the participants regarding whether they had heard about the Nipah virus or not.

Among the 2121 participants, 1472 (69.38 %) reported having heard about the NiV, while 649 individuals (30.62 %) stated that they had not heard about it at all [Fig. 1]. Participants primarily acquired knowledge about the virus from various sources, with social media (29.9 %) being the main one, followed by television (25.41 %) [Table 2]. Educational institutions also played a significant role, with 18.95 % of participants gaining knowledge from this source. Friends and workplaces were less common sources, contributing 6.39 % and 5.91 %, respectively.

**Table 2:** Source of knowledge for the participants who had knowledge of Nipah Virus.

Source of knowledge	Frequency (n = 1472)	Percentage (%)
Educational institution	279	18.95
Friends	94	6.39
Newspaper	201	13.65
Social media	437	29.69
TV	374	25.41
Workplace	87	5.91

**Fig. 2:**
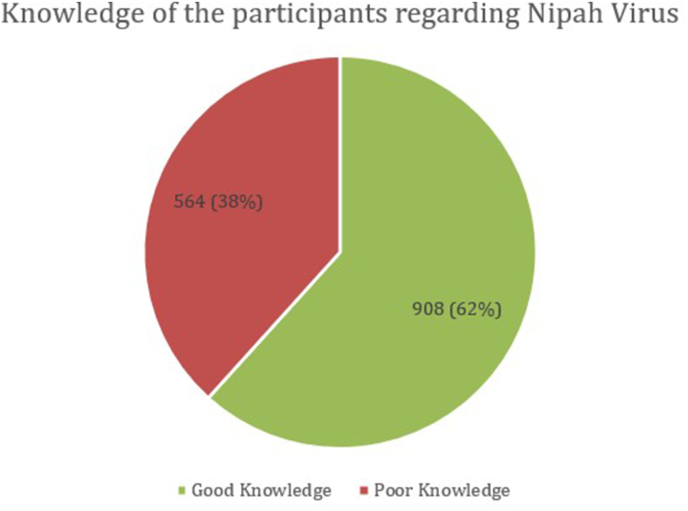
Knowledge of the participants (n = 1472) Regarding Nipah virus.

Among the participants surveyed, 908 individuals (62 %) demonstrated good knowledge about NiV, while 564 individuals (38 %) had poor knowledge [Fig. 2].

In the univariate analysis [Table 3], individuals aged 31–40 years showed notably lower odds of poor knowledge compared to the 21–30 years group (OR = 0.80, 95 % CI: 0.61–1.06, p < 0.01), while other age groups did not show significant associations. Gender-wise, females showed slightly higher odds of poor knowledge, though not statistically significant. Notably, lower education levels, including no formal education (OR = 5.47, 95 % CI: 1.94–15.47, p < 0.01), primary education (OR = 4.81, 95 % CI: 2.24–10.32, p < 0.01), and secondary education (OR = 1.80, 95 % CI: 1.20–2.72, p < 0.01), were strongly associated with poor knowledge. Similarly, various occupations such as students (OR = 1.93, 95 % CI: 1.26–2.98, p < 0.01), housewives (OR = 2.12, 95 % CI: 1.32–3.39, p < 0.01), and individuals engaged in business (OR = 2.18, 95 % CI: 1.34–3.56, p < 0.01) demonstrated significantly higher odds of poor knowledge compared to healthcare workers. Geographically, several divisions like Rangpur (OR = 0.51, 95 % CI: 0.32–0.80, p < 0.01), Khulna (OR = 1.95, 95 % CI: 1.32–2.89, p < 0.01), Mymensingh (OR = 3.00, 95 % CI: 1.88–4.81, p < 0.01), and Sylhet (OR = 1.19, 95 % CI: 0.79–1.79, p = 0.41) had significantly lower odds of poor knowledge compared to Dhaka.

**Table 3:** Univariate logistic regression analysis exploring factors associated with poor knowledge about the Nipah virus among participants.

Variables	Odds Ratio	95 % Confidence Interval		p-value
		low	high	
Age (Years)				
21–30	Ref			
≤20	1.20	0.82	1.74	0.34
31–40	0.80	0.61	1.06	0.12
41–50	1.20	0.86	1.67	0.29
>50	1.34	0.90	1.98	0.14
**Gender**				
Male	Ref			
Female	1.12	0.90	1.38	0.29
**Education**				
Post graduate	Ref			
No formal education	5.47	1.94	15.47	**<0.01**
Primary	4.81	2.24	10.32	**<0.01**
Secondary	1.80	1.20	2.72	**<0.01**
Higher secondary	0.94	0.69	1.29	0.71
Graduate	0.89	0.65	1.21	0.44
**Occupation**				
Healthcare worker	Ref			
Student	1.93	1.26	2.98	**<0.01**
Housewife	2.12	1.32	3.39	**<0.01**
Service and Job Holders	2.03	1.30	3.19	**<0.01**
Business	2.18	1.34	3.56	**<0.01**
Others	3.60	2.00	6.48	**<0.01**
**Division**				
Dhaka	Ref			
Chittagong	1.02	0.73	1.42	0.91
Rangpur	0.51	0.32	0.80	**<0.01**
Rajshahi	1.48	1.04	2.10	**0.03**
Khulna	1.95	1.32	2.89	**<0.01**
Mymensingh	3.00	1.88	4.81	**<0.01**
Sylhet	1.19	0.79	1.79	0.41
Barishal	0.81	0.51	1.30	0.38
**Source ofknowledge**				
Educational institution	Ref			
Social media	2.27	1.63	3.17	**<0.01**
Friends	2.80	1.72	4.57	**<0.01**
Newspaper	1.22	0.81	1.84	0.34
TV	2.32	1.66	3.23	**<0.01**
Workplace	1.70	1.02	2.83	**0.04**

**Fig. 3:**
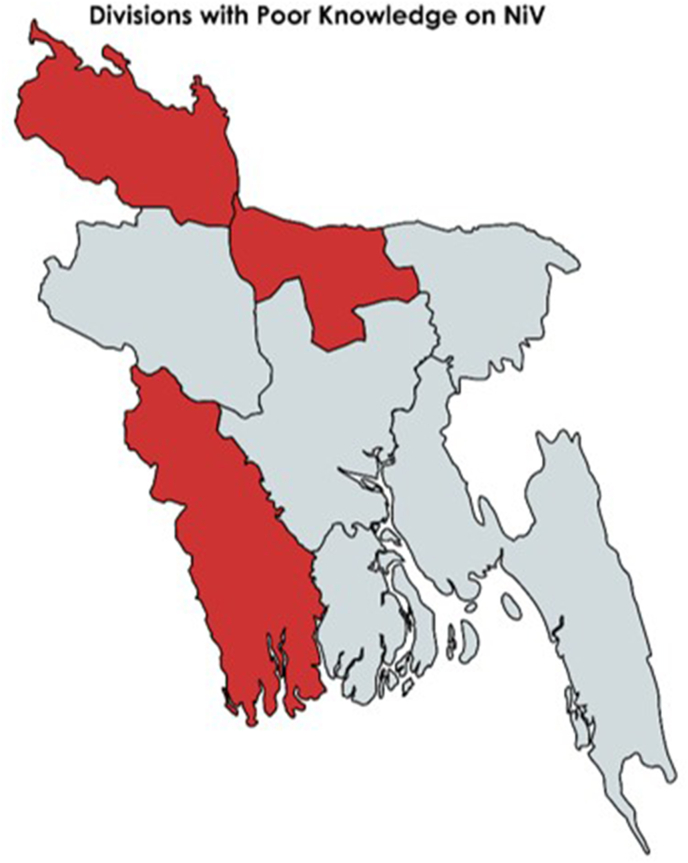
Divisions of Bangladesh with Poor Knowledge of the Nipah Virus. **∗**Red marked areas include Mymensingh, Khula and Rangpur Divisions that show poor knowledge on both univariate and multivariate logistic regression analysis.

Table 4 presents the results of the multivariate logistic regression analysis investigating factors associated with poor knowledge about the NiV among participants. Participants aged 31–40 years showed 43 % lower odds of having poor knowledge compared to those aged 21–30 years (OR = 0.57, 95 % CI: 0.39–0.82, p < 0.01) which is significant. Females showed 1.38 times higher odds of poor knowledge compared to males (OR = 1.38, 95 % CI: 1.05–1.81, p = 0.02). Individuals with no formal education (OR = 5.21, 95 % CI: 1.74–15.61, p < 0.01), primary education (OR = 4.10, 95 % CI: 1.76–9.51, p < 0.01), and secondary education (OR = 1.90, 95 % CI: 1.14–3.16, p = 0.01) demonstrated significantly higher odds of poor knowledge compared to those with post-graduation qualifications. Students (OR = 1.93, 95 % CI: 1.19–3.16, p = 0.01), individuals engaged in business (OR = 2.25, 95 % CI: 1.25–4.05, p = 0.01), and those categorised under other occupations (OR = 2.48, 95 % CI: 1.28–4.84, p = 0.01) displayed significantly higher odds of poor knowledge compared to healthcare workers. Geographically, several divisions such as Rangpur (OR = 0.43, 95 % CI: 0.26–0.70, p < 0.01), Khulna (OR = 1.70, 95 % CI: 1.10–2.61, p = 0.01), and Mymensingh (OR = 2.77, 95 % CI: 1.70–4.53, p < 0.01) exhibited significantly lower odds of poor knowledge compared to Dhaka [Fig. 3]. Obtaining knowledge from social media (OR = 2.29, 95 % CI: 1.61–3.26, p < 0.01), friends (OR = 2.36, 95 % CI: 1.39–4.01, p < 0.01), and television (OR = 2.25, 95 % CI: 1.52–3.33, p < 0.01) were associated with significantly higher odds of poor knowledge compared to educational institutions.

**Table 4:** Multivariate logistic regression analysis exploring factors associated with poor knowledge about the Nipah virus among participants.

Variables	Odds Ratio	95 % Confidence Interval		p-value
		low	high	
Age (Years)				
21–30	Ref			
≤20	0.99	0.63	1.55	0.97
31–40	0.57	0.39	0.82	**<0.01**
41–50	0.80	0.52	1.24	0.31
>50	0.76	0.46	1.23	0.27
**Gender**				
Male	Ref			
Female	1.38	1.05	1.81	**0.02**
**Education**				
Post Graduate	Ref.			
No formal education	5.21	1.74	15.61	**<0.01**
Primary	4.10	1.76	9.51	**<0.01**
Secondary	1.90	1.14	3.16	**0.01**
Higher secondary	0.85	0.58	1.26	0.43
Graduation	0.86	0.60	1.22	0.40
**Occupation**				
Healthcare worker	Ref.			
Student	1.93	1.19	3.16	**0.01**
Housewife	1.49	0.83	2.69	0.18
Service and job holders	2.20	1.35	3.60	**<0.01**
Business	2.25	1.25	4.05	**0.01**
Others	2.48	1.28	4.84	**0.01**
**Division**				
Dhaka	Ref.			
Chittagong	1.06	0.75	1.51	0.76
Rangpur	0.43	0.26	0.70	**<0.01**
Rajshahi	1.34	0.92	1.94	0.14
Khulna	1.70	1.10	2.61	**0.01**
Mymensingh	2.77	1.70	4.53	**<0.01**
Sylhet	1.36	0.87	2.14	0.17
Barishal	0.89	0.54	1.45	0.63
**Source of Knowledge**				
Educational institution	Ref.			
Social media	2.29	1.61	3.26	**<0.01**
Friends	2.36	1.39	4.01	**<0.01**
Newspaper	1.23	0.80	1.9	0.36
TV	2.25	1.52	3.33	**<0.01**
Workplace	1.90	1.05	3.41	**0.03**

## 4 Discussion

Our nationwide study presents valuable insights on the knowledge of NiV among participants in Bangladesh. The proportion of respondents demonstrating good knowledge of NiV stands at 62 %, whereas 38 % showedpoor knowledge, indicating areas for improvement in the existing educational initiatives. Meanwhile a study conducted in Saudi Arabia reflected about 45 % Healthcare workers had poor knowledge of NiV.^24^ Prior to the Covid era, studies showed that because of a lack of knowledge, people were unable to take advantage of techniques available, resulting in frequent outbreaks of NiV infections among community members in several parts of Bangladesh every year.^25^

This study reveals several factors associated with both high and poor knowledge levels of the NiV among participants, shedding light on crucial aspects that influence public health literacy in Bangladesh. Firstly, age emerges as a significant determinant, with participants aged 31–40 years being more likely to possess good knowledge of NiV compared to younger individuals which aligns with a similar study in Bangladesh conducted on a smaller population by Hassan et al. (2020) where older age was significantly associated with having a better knowledge of NiV.^21^ This finding suggests that older age groups may have accumulated more experience or exposure to information over time, contributing to a better knowledge retention of NiV. Additionally, it raises concerns about potential negligence among younger age groups regarding the risks associated with consuming raw date sap. Regretfully, however, a different nationwide knowledge survey conducted in Bangladesh found that only over a quarter of participants had heard of a disease caused by consuming raw sap.^26^

Gender also plays a role in our study with females showing slightly higher odds of poor knowledge compared to males. This observation is in contradiction with a study conducted in South India on knowledge, attitude and practice of NiV among healthcare workers (e.g., doctors, nurses, paramedical staff) where they found that female respondents were more likely to have good knowledge NiV than males.^15^ Further exploration into gender-related differences in health-seeking behaviors and information source treatment seeking behaviors associated with various epidemiological factors particularly socio-cultural barriers, could provide valuable insights for tailored educational interventions. Education level emerges as a robust predictor of NiV knowledge in our study, reflecting a significant disparity in awareness among different educational strata.^27^ This finding underscores the pivotal role of education in shaping health knowledge and behaviors, especially in the Bangladesh context, where socioeconomic factors often intersect with educational opportunities.^28^ This finding aligns with the findings of Hassan et al. (2020),^21^ where similar occupational groups showed poor knowledge of NiV.

Geographic location is another important determinant, with several divisions such as Rangpur, Khulna, and Mymensingh exhibiting significantly lower odds of poor knowledge compared to Dhaka. Since its first outbreak in 2001, Bangladesh has witnessed annual seasonal outbreaks of NiV infection, particularly in regions including Narsingdi, Rajbari, Shariatpur, Naogaon, Natore, Pabna, and Rajshahi, which witnessed higher case fatality rates.^29^ This is particularly alarming because ‘Rajshahi’ is considered to be an area of Bangladesh at risk but seemed to have ‘poor’ knowledge in our study. This contrasts with findings from a previous study by Hassan et al. (2020),^21^ which specifically focused on Nipah outbreak areas (NOAs) and non-NAOs (NNOAs) within districts like Faridpur, Manikganj and Rajshahi. The observed contrast suggests that factors beyond geographical proximity to outbreak areas may influence knowledge levels, highlighting the complexity of regional differences in NiV awareness. Another study found less knowledge among people of Chittagong compared to Dhaka city.^30^ Despite these differences, both studies emphasise the importance of targeted interventions tailored to regional needs and priorities, emphasising the significance of understanding local socio-cultural factors and stigma in designing effective public health campaigns and surveillance strategies. We stress the importance of targeted surveillance and intervention strategies in high-risk regions such as Rajshahi to curb future outbreaks effectively.

In our study, the observed higher odds of poor knowledge among participants who acquired information from sources like social media and television compared to those relying on educational institutions are particularly concerning. However, this result contradicts another study that compared a broader Nepalese sample, showing that respondents who learnt about the monkeypox virus and its epidemic via social media had considerably greater knowledge.^31^ During the AI era, in comparison to other forms of media, newspapers, local governments, and healthcare practitioners tend to be less common providers of disease information. This highlights the potential drawbacks of relying solely on social media for public health education and awareness campaigns.

The strengths of our study include its nationwide scope, providing a comprehensive understanding of NiV knowledge across Bangladesh. Additionally, the study boasts a representative and large sample size, proportionately distributed among divisions, based on census data, ensuring the generalizability of findings to the broader population. Furthermore, the comprehensive data analysis conducted enables thorough exploration of factors influencing levels of NiV knowledge. However, several limitations must be acknowledged. Firstly, the cross-sectional nature of the study prevents any causal associations between variables. Secondly, the use of convenience sampling may introduce selection bias, limiting the generalizability of results. Lastly, while the study provides quantitative insights, the lack of qualitative data limits the depth of understanding regarding the nuances of NiV knowledge and associated factors among participants.

## 5 Conclusion

In conclusion, to our knowledge, this is the first ever nationwide study on NiV awareness of the general population in Bangladesh. Overall, our findings underscore the multifaceted nature of factors influencing NiV knowledge among the Bangladeshi population. By addressing knowledge gaps and promoting health literacy, we can enhance its prevention and control efforts, ultimately reducing the burden of this infectious disease on public health in Bangladesh and beyond. Apart from these, this study results highlight the importance of multiple platforms for NiV education and awareness campaigns in the future and strengthening the participation of community leaders and healthcare workers.

## CRediT authorship contribution statement

**Mobin Ibne Mokbul:** Writing – original draft, Visualization, Supervision, Project administration, Methodology, Investigation, Formal analysis, Data curation, Conceptualization. **Shuvajit Saha:** Writing – review & editing, Methodology, Investigation, Data curation. **Samiha Nahar Tuli:** Writing – original draft, Methodology. **Fatema Binte Nur:** Writing – original draft. **A.M. Khairul Islam:** Writing – review & editing, Supervision, Conceptualization. **Tariful Islam:** Methodology, Data curation. **Shirsho Shreyan:** Data curation. **Alok Bijoy Bhadra:** Data curation. **Golam Dastageer Prince:** Data curation. **Irfath Sharmin Eva:** Data curation. **Mustari Nailah Tabassum:** Data curation. **Ferdous Wahid:** Data curation. **Md Irfan Bin Kayes:** Data curation. **Nazim Hassan Ziad:** Data curation. **Mohammad Delwer Hossain Hawlader:** Writing – review & editing, Supervision.

## Ethical approval

Ethical approval was obtained from the Institutional Review Board (IRB)/Ethical Review Committee (ERC) of North South University, Bangladesh (IRB approval #2022IOR-NSU/lRB/1204).

## Consent

Each participant consented to participate in the study.

## Funding

The authors did not receive any funding.

## Declaration of competing interest

The authors declare no conflict of interest.

## Data Availability

Data will be made available on request.

## Abbreviations

**NiV**: Nipah Virus
**HCWs**: Healthcare Workers
**COVID-19**: Coronavirus Disease 2019
**RNA**: Ribonucleic Acid
**NOAs**: Nipah Outbreak Areas
**NNOAs**: Non-Nipah Outbreak Areas
**MPXV**: Monkeypox Virus
**AI**: Artificial Intelligence
**OR**: Odds Ratio
**CI**: Confidence Interval
